# Millstone: software for multiplex microbial genome analysis and engineering

**DOI:** 10.1186/s13059-017-1223-1

**Published:** 2017-05-25

**Authors:** Daniel B. Goodman, Gleb Kuznetsov, Marc J. Lajoie, Brian W. Ahern, Michael G. Napolitano, Kevin Y. Chen, Changping Chen, George M. Church

**Affiliations:** 1 Department of Genetics, Harvard Medical School, Boston, MA USA; 2 Wyss Institute for Biologically Inspired Engineering, Harvard Medical School, Boston, MA USA; 3 Program in Biophysics, Harvard University, Boston, MA USA; 4 Massachusetts Institute of Technology, Cambridge, MA USA; 5 Biological and Biomedical Sciences, Harvard Medical School, Boston, MA USA

**Keywords:** Synthetic biology, Bioinformatics, Synthetic genomics, Genome engineering, Microbial evolution, Whole-genome sequencing, Laboratory evolution, Experimental evolution

## Abstract

Inexpensive DNA sequencing and advances in genome editing have made computational analysis a major rate-limiting step in adaptive laboratory evolution and microbial genome engineering. We describe Millstone, a web-based platform that automates genotype comparison and visualization for projects with up to hundreds of genomic samples. To enable iterative genome engineering, Millstone allows users to design oligonucleotide libraries and create successive versions of reference genomes. Millstone is open source and easily deployable to a cloud platform, local cluster, or desktop, making it a scalable solution for any lab.

**Electronic supplementary material:**

The online version of this article (doi:10.1186/s13059-017-1223-1) contains supplementary material, which is available to authorized users.

## Background

Microbial populations can harbor a staggering amount of genomic diversity, enabling them to evolve and adapt to diverse environments. Adaptive laboratory evolution uses this process to generate strains that are useful for biotechnology or for answering fundamental biological questions [1]. In addition to harnessing natural variation, biologists can generate targeted genomic diversity in a population of cells and then screen or select for phenotypes of interest [2]. The decreasing cost of reading and writing microbial genomes has made it possible to generate billions of combinatorial genomic variants per day at specific loci [2–4] and to sequence entire *Escherichia coli* genomes for less than 25 USD per sample [5, 6] (Additional file 1: Note S1).

Computational analysis is increasingly a bottleneck when mapping whole-genome data to phenotypes across many samples. Going from raw DNA sequencing reads to annotated variants requires the integration of a large number of disparate tools, usually assembled into an ad hoc pipeline by individual labs and followed by time-intensive manual confirmation of variants. There remains a critical need for an integrated solution capable of comparative analysis among multiple genomes and supporting interactive querying and data visualization, collaboration, genome versioning, and the design of additional mutations or reversions (Additional file 1: Table S1; Note S2).

**Fig. 1:**
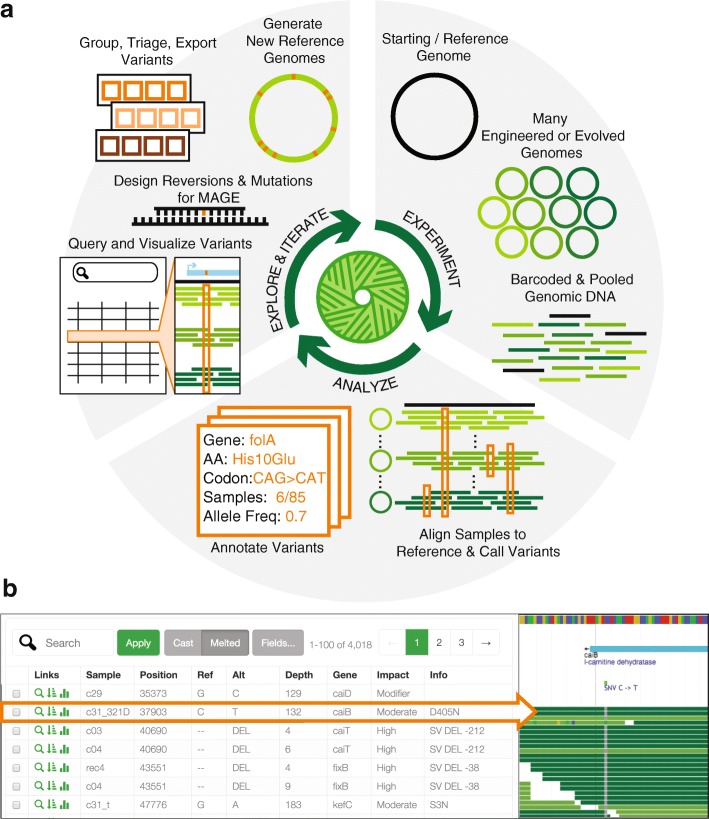
Millstone enables rapid iterative multiplex genome analysis and engineering. **a** To use Millstone, a researcher uploads a reference genome and next-generation sequencing reads for many individual genomic clones, for example from long-term evolution or targeted genome editing. Millstone performs alignment and variant calling for both single nucleotide variants and structural variation and then assigns predicted effects based on reference genome annotations. A unified data model stores sample genotype, phenotype, and variant annotation data. Variants can then be queried, filtered, and grouped into sets for export, triage, and analysis. These variant sets can be used to design oligonucleotides to recreate or revert mutations of interest, or used to generate new versions of the reference genome. **b** A combined screenshot of the Millstone analysis and alignment visualization views (condensed and cropped for clarity). A custom query language and a corresponding query form in the user interface allow searching and filtering over the data. As variant calls sometimes require visual inspection and comparison, Millstone’s variant analysis view provides programmatically generated links to visualizations of the relevant read alignments in JBrowse [18]

To address this need, we developed Millstone, a web-based software platform that supports an iterative process of multiplex mutation analysis and genome engineering. Millstone automates read alignment and variant calling using a hybrid reference-based and de novo assembly approach. It allows researchers to explore and compare mutations among genomic samples, and finally it creates updated reference genomes and designs new genomic edits for subsequent rounds of experiments (Fig. 1 a). Serving as both a genomics pipeline and a platform for exploring whole-genome sequencing data, Millstone provides a powerful user-friendly interface that allows researchers to investigate individual variants through interactive filtering and alignment visualization (Fig. 1 b). Teams of researchers can simultaneously and securely access a single Millstone instance for data sharing and collaboration.

## Results

**Fig. 2:**
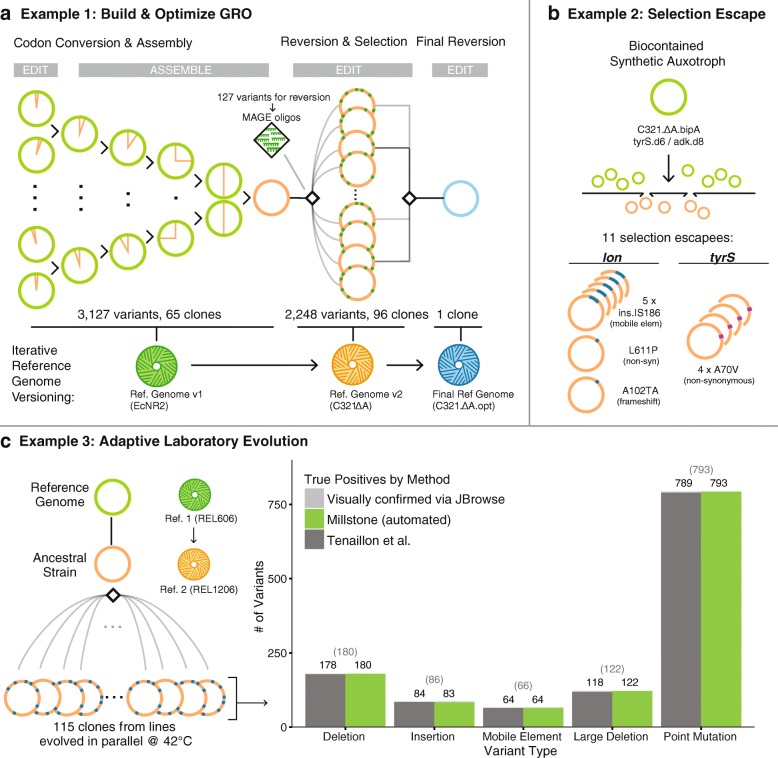
Millstone accurately detects genomic variants and can iteratively version genomes. **a** Millstone was used to analyze genomic clones involved in generating and rationally optimizing a genomically recoded organism. MAGE [2] and CAGE [3] were used to generate the C321. *Δ*A strain of *Escherichia coli* [7]. With sequencing data from these strains, Millstone confirmed the designed mutations, identified and annotated off-target mutations, and generated a new reference genome. Further reversion of variants was performed with MAGE to improve the strain’s fitness [8], and a final reference genome was generated. **b** Analysis of 11 escapee clones from a biocontainment selection with a synthetic non-standard amino acid (nsAA) auxotrophy [9] identified two escape mechanisms, either mutation of *tyrS* or disruption of *lon*. **c** Millstone can also be used for adaptive laboratory evolution studies. We employed Millstone to analyze mutations across 115 clones in the Tenaillon et al. [10] high-temperature evolution experiment. Millstone was used to create a new reference genome for the ancestral strain from REL606, the closest available reference genome, and called variants against this new reference. Millstone reports 99.2% of SNVs, deletions, and mobile elements found by the Tenaillon pipeline, as well some not identified in the original study (Additional file 1: Table S2). *GRO* genomically recoded organism, *Ref.* reference, *SNV* single-nucleotide variant

Millstone was built in response to challenges encountered during the construction of the genomically recoded organism (GRO) C321. *Δ*A [7], a strain of *E. coli* in which all 321 UAG stop codons were replaced with a synonymous UAA. Multiplex automated genome engineering [2] (MAGE) was used to introduce sets of ten mutations into 32 strains. Conjugative assembly genome engineering [3] (CAGE) was used to hierarchically combine redesigned regions into a chromosome with all 321 UAGs recoded (Fig. 2 a, green). We sequenced 68 intermediate clones to confirm the designed changes but our initial analyses were slow, error-prone, and lacked the ability to visualize and compare evidence for mutations among samples. Millstone solved these issues, allowing us to identify and track the 3127 designed and off-target mutations across all strains. Finally, by iteratively applying mutations directly to the initial reference genome and re-aligning reads, Millstone allowed us to generate a new C321. *Δ*A reference sequence that incorporated 355 additional off-target mutations that had accumulated during strain construction (Fig. 2 a, green and orange).

Millstone’s ability to generate clonal genotypes rapidly from whole-genome sequencing reads enabled a follow-up project to improve the fitness of the GRO. The final strain from Lajoie et al. [7] demonstrated incorporation of proteins containing non-standard amino acids (nsAAs), but suffered from an impaired growth phenotype, which we hypothesized was due to a subset of the 355 off-target mutations. We developed an iterative method for systematically optimizing strain fitness through predictive modeling and multiplex testing of reversions [8]. Millstone was used throughout this process: first, to rank high-effect candidates for reversion, then to design oligonucleotides for MAGE, and finally to report variants from whole-genome sequencing of 96 edited clones (Fig. 2 a, orange). Once the final subset of effective reversions was identified and used to construct a faster-growing GRO, Millstone was also used to produce a final reference genome for the improved strain.

Millstone’s de novo assembly and genotype comparison features were crucial in a project to engineer a biocontained version of the GRO that is dependent on an nsAA for survival [9]. A major challenge in engineering biocontainment, and in selection more generally, is diagnosis of escape mechanisms. In Mandell et al. [9], plating of early versions of the biocontained GRO on non-permissive media revealed individual clones that could survive without the essential nsAA. We performed whole-genome sequencing on 11 escapee clones and several controls and used Millstone to identify loci enriched for mutations across escapees. This led to the discovery and validation of two primary mechanisms of escape: a single off-target nonsynonymous mutation in the redesigned *tyrS* gene occurring in 4/11 clones and disruption of the *lon* protease in the remaining seven clones. Millstone revealed several modes of *lon* disruption: a frameshift (1/7), nonsynonymous substitution (1/7), and insertion of a mobile element upstream of the gene (5/7) (Fig. 2 b). To identify and map these mobile elements and other structural variants precisely, Millstone combines a local de novo re-assembly approach with coverage-based deletion calling (Additional file 1: Figure S3). Rapid analysis of escapee clones allowed us to identify and validate the key mechanisms of escape from biocontainment, so that further modifications lowered escape rates by at least 5 orders of magnitude (undetectable escape with detection limit 2.2×10^−12^ escapees per cfu) [9].

Millstone can also be used to analyze genomic variation in samples undergoing adaptive laboratory evolution. In Tenaillon et al. [10], 115 lines of *E. coli* were grown at 42^∘^C in parallel for over 2000 generations in an attempt to identify convergent evolutionary responses to this environmental challenge (Fig. 2 c). This impressive effort required a custom sequencing analysis pipeline consisting of over half a dozen tools, followed by manual validation and visual confirmation of all 1331 variants. We reanalyzed the raw data from this project in Millstone and identified 99.7% of single-nucleotide variants (SNVs) and 98.9% of structural variants and mobile element insertions. Millstone further discovered eight SNVs, four large deletions, and two mobile element insertions that were not reported in the original work (Fig. 2 c, Additional file 1: Table S2). On an Amazon Web Services (AWS) EC2 instance, the entire process from sample upload to variant triage across all 115 strains took a single day (Additional file 1: Table S3).

## Conclusions

New technologies for constructing, screening, and selecting microbial genomes now allow for increasingly complex functional genomics studies and bioengineering endeavors. As the sequence constraints of the genome come into focus, the promise of designing new organisms to address medical and material challenges is becoming a reality [11]. The path forward requires rapid construction and characterization of successive versions of redesigned genomes [12, 13], and computational genome design and analysis tools will increasingly become integral to this process. Researchers who already have raw sequencing data can use Millstone to identify and explore mutations. We have reduced the barrier for other labs to use Millstone by making the software deployable on AWS. Documentation, detailed instructions for launching an AWS instance with the latest Amazon Machine Image, and an online demo are available at http://churchlab.github.io/millstone.

## Methods

### Deployment via AWS

Using Millstone via AWS is the recommended option for most users. We have preconfigured a Millstone installation as an Amazon Machine Image, allowing users to sidestep all dependency installation and configuration steps. Researchers can provision a fully configured private instance of Millstone running on AWS in minutes and can specify compute, memory, and disk requirements to match project needs. AWS allows resizing of a machine without losing data. For most projects and benchmarking, we use a 32-core r3.8xlarge instance type for alignment and variant calling, and then resize the machine to the two-core c3.large or c4.large instance type for data exploration. Academic labs can apply for the AWS Cloud Credits for Research program (https://aws.amazon.com/grants/).

### Analysis pipeline

Millstone provides a user interface that guides a researcher through uploading a reference genome (Genbank or FASTA) and Illumina sequencing reads (FASTQ). During the sample upload procedure, users can also include additional per-sample phenotype information such as growth rate, sample type, and relationships to other strains (e.g., parent/child relationships across conjugation, assembly, or evolution). In the background, Millstone checks the quality of the input FASTQ files via FASTQC [14] and provides a link to the report. The Millstone analysis pipeline then performs alignment, variant-calling, and annotation of called variants. Millstone aligns reads using BWA-MEM [15], parallelizing across available cores. Millstone calls SNVs using Freebayes [16] and structural variants using a custom contig assembly-based method (see “De novo assembly pipeline” below) and a custom coverage-based method. Freebayes takes into account individual base quality scores to estimate variant quality. A diagram of the pipeline and the parallelization scheme is shown in Additional file 1: Figure S1. Even though Millstone was primarily designed for haploid bacterial genomes, the default and recommended mode for calling SNVs in Millstone is diploid. This allows reporting of marginal calls that are neither clean wild-type nor clean mutant alleles, indicated as “heterozygous” under the GT_TYPE field (GT in VCF format). If the user-provided reference genome is annotated (i.e., Genbank format), the Millstone analysis pipeline can annotate variants with the predicted effect using SnpEff [17].

### Variant exploration view

Millstone provides an interactive user interface for exploring mutations and comparing genomes. Two primary data view modes, *cast* and *melted*, allow the exploration of data with samples aggregated by mutation or by individual mutation-sample relationships, respectively. The terms “cast” and “melt” are taken from the R programming language, and are the the same as operations referred to variously as “pivot/unpivot”, “pivot/melt”, and “wide/long” in different data manipulation environments. Each mutation row includes icons that link to the relevant view of aligned reads in JBrowse. This is useful for performing visual quality control on aligned regions to verify that reads are properly aligned around a variant or to diagnose complex structural events. Visual quality control is particularly important for inspecting marginal variant calls (see “Analysis pipeline”), where only a fraction of aligned reads show a SNV. These can indicate regional duplication, non-unique mapping of reads, or non-clonality.

A query language allows filtering of variants according to fields such as read depth, evidence quality, gene affected, and predicted impact (online documentation). The filter key GT_TYPE is particularly important with respect to identifying mutant alleles and distinguishing them from marginal calls. GT_TYPE can take on values of 0 (strong evidence for a wild-type allele), 1 (marginal call), or 2 (strong evidence for a mutant allele). The query language allows for Boolean combinations of key-value statements. For example, the following query filters the displayed variants down to only well-supported mutants that have a moderate to strong effect on some gene:

GT_TYPE = 2 & (INFO_EFF_IMPACT = HIGH | INFO_EFF_IMPACT = MODERATE)

This query language is implemented in the user interface as a search form, in which previously run queries can be saved. Further information and examples of queries can be found in the online documentation (http://millstone.readthedocs.io/).

### Visualizing alignments

Millstone uses JBrowse [18] to visualize read alignments, enabling manual quality control and verification of complex structural events. For each called variant, Millstone programmatically generates a link that brings up JBrowse at the affected locus showing the relevant alignment tracks. In the *cast* view, multiple JBrowse tracks will be shown simultaneously if the variant is present in multiple samples.

### Variant sets

Variant sets are an important unit of operation in Millstone and they allow grouping of variants after filtering. Millstone’s analysis pipeline also allocates variants to several common variant sets by default, including sets indicating insufficient coverage, no coverage, greater than expected coverage, or poor mapping quality (corresponding perhaps to regions that are not unique).

Variant sets can also be used to generate oligonucleotides targeting or reverting the mutations in the set, via an integrated Python implementation of optMAGE [2] (https://github.com/churchlab/optmage). Millstone’s genome versioning feature allows for variant sets to be used to generate a new version of the reference genome containing those variants as ref alleles. In particular, we show the use of this feature to generate updated reference genomes for the C321. *Δ*A strain and its improved version, C321. *Δ*A.opt.

### De novo assembly pipeline

After Illumina sequencing reads are aligned to the genome, Millstone identifies candidate reads that may indicate structural variants, including unmapped, clipped, and split reads, as well as their pairs. These reads may indicate the presence of complex structural events such as deletions, novel sequence insertions, and translocations of mobile elements. Velvet [19] is used to assemble these reads into de novo contigs.

Once the reads are assembled into contigs by Velvet, those contigs over a size threshold are aligned back to the reference genome using BWA-MEM [15]. These contig-to-reference alignments are used by Millstone to generate a graph whose nodes are contigs and reference sequence fragments and whose edges are alignments. Individual contigs and their reference junctions can be browsed and downloaded by the user. The contig sequences can also be downloaded as FASTA records and blasted individually by the user. Contigs whose edges map to annotated mobile elements are also labeled in the user interface. Novel sequence and mobile element insertions consist of multiple graph edges (two edges for a novel sequence and four for mobile element insertions; Additional file 1: Figure S3b). These variants are identified by a graph-traversal algorithm and converted into VCF records, which are added to the variant database.

### Tenaillon et al. variant comparison

Raw data for Tenaillon et al. [10] was downloaded from: http://wfitch.bio.uci.edu/~tdlong/PapersRawData/Tenaillon.rawdata.tar.gz. Using the strain metadata provided with the sequencing data, we generated a targets file containing fitness ratios, line labels, and paths to read files for all 115 samples.

We first used Millstone to align the ancestor strain (line 0) to the REL606 reference genome (NCBI accession CP000819). This found a 6.93 kb deletion in *scgB* (3,894,998 bp) and an IS186 insertion near *fimA* (4,517,603 bp). We used Millstone’s reference genome versioning feature to generate the REL1206 reference genome by applying these structural events to REL606.

Finally, all 115 samples (including the ancestor strain) were realigned to the new REL1206 genome using Millstone. The data were exported to CSV format for comparison with the variants called by Tenaillon et al. For a list of mutation discrepancies between Millstone and Tenaillon, see Additional file 1: Table S2.

### Optimizing genomically recoded organism C321. *Δ*A

Millstone was used to create a new reference genome for the final C321. *Δ*A strain. Mutations called by Millstone were triaged based on quality and added to a variant set. Then, from the variant set view, the generate new reference genome feature was used to create the new genome. This process was iterated two additional times until there were no structural or SNVs called against the new final reference genome.

Millstone includes a SnpEff integration [17] that allows the annotation of mutations with predicted effects. We used Millstone to generate 127 MAGE oligos to revert mutations predicted to have strong fitness effects.

After sequencing 96 clones, which underwent from 5 to 50 cycles of MAGE with subsets of these 127 oligos, Millstone was used to align and call variants across all clones. This process took 3 hours on a 32-core machine (Additional file 1: Table S3). Millstone generated a CSV file reporting evidence for variants, and this was combined with per-strain doubling time measurements to generate a regularized linear model [8]. Additionally, during modeling and iterative testing, we returned to Millstone to use the JBrowse view to visually validate and compare marginal variant calls among samples.

Finally, Millstone was used to align, call variants, and produce a reference sequence for the final optimized strain, which contained six reverted alleles and nine de novo mutations relative to the starting C321. *Δ*A strain.

### Mandell et al. variant comparison

We obtained sequencing reads from the authors [9] and uploaded and aligned them to the C321. *Δ*A genome (described above). In addition to the 11 escapee genomes on the adk.d8 and tyrS.d6 strain backgrounds, we also sequenced control clones for each background, which remained dependent on the nsAA bipA, and some additional escapee clones on other nsAA-dependent backgrounds.

To identify the SNVs, including the *lon* and *tyrS* A70V mutations, we used Millstone to look for non-designed mutations that occurred only in the escapee strains and not in the bipA-dependent strains. To exhaustively identify transposon insertions where only partial alignment support was available, we additionally downloaded the raw contig list from Millstone and looked for contigs with graph edges mapping from mobile elements to other genome locations. We identified junction contigs that bridge the IS186 mobile element and the *lon* gene in five strains (Additional file 1: Figure S3).

### Data representation

Millstone stores variant, experiment sample, and evidence data across several tables in a PostgreSQL database (Additional file 1: Figure S2). Returning results for user queries from the variant exploration view requires a join operation among multiple tables, which is expensive and does not scale well. To address this, we use PostgreSQL’s materialized view feature to compute and store a single denormalized table with all variant-sample evidence. Subsequent queries can then be performed directly against this table. Benchmarking on an AWS EC2 c3.large (two-core 3.75 GB RAM), a dataset with 100 samples and 2500 variants required 1 min 44 seconds to compute a materialized view and typical queries required less than 1 s. The Millstone database only recomputes the materialized view when the underlying data has changed.

## Availability and requirements

The Millstone source code is available at: https://github.com/churchlab/millstone. The software is provided under the MIT license, available at: https://github.com/churchlab/millstone/blob/master/LICENSE. Documentation is available at http://millstone.readthedocs.io/.

## Additional file

Additional file 1 Supplementary figures, notes, and tables. (PDF 2219 kb)
