# Supplementary material for: Millstone: software for multiplex microbial genome analysis and engineering
Source: Genome Biol. 2017 May 25;18:101. doi: 10.1186/s13059-017-1223-1 (PMC5445467; doi:10.1186/s13059-017-1223-1)
Supplement: Additional file 1 — Supplementary figures, notes, and tables. (PDF 2219 kb) [file 13059_2017_1223_MOESM1_ESM.pdf]

Supplementary Figures, Notes, and Tables for Millstone: software for multiplex microbial genome analysis and engineering

Daniel B. Goodman[1,2,6], Gleb Kuznetsov[1,2,3,6], Marc J. Lajoie[1,2], Brian W. Ahern[5], Michael G. Napolitano[1,2,4], Kevin Y. Chen[5], Changping Chen[5], and George M. Church[1,2,7]

[1]Department of Genetics, Harvard Medical School, Boston, Massachusetts, USA

[2]Wyss Institute for Biologically Inspired Engineering, Harvard Medical School, Boston, Massachusetts, USA

[3]Program in Biophysics, Harvard University, Boston, Massachusetts, USA

[4]Biological and Biomedical Sciences, Harvard Medical School, Boston, Massachusetts, USA

[5]Massachusetts Institute of Technology, Cambridge, Massachusetts, USA

[6]These authors contributed equally to this work.

[7]Correspondence should be addressed to G.M.C. (gchurch@genetics.med.harvard.edu).

Supplementary Figures S1-S3

Supplementary Tables S1-S3

Supplementary Notes 1-2

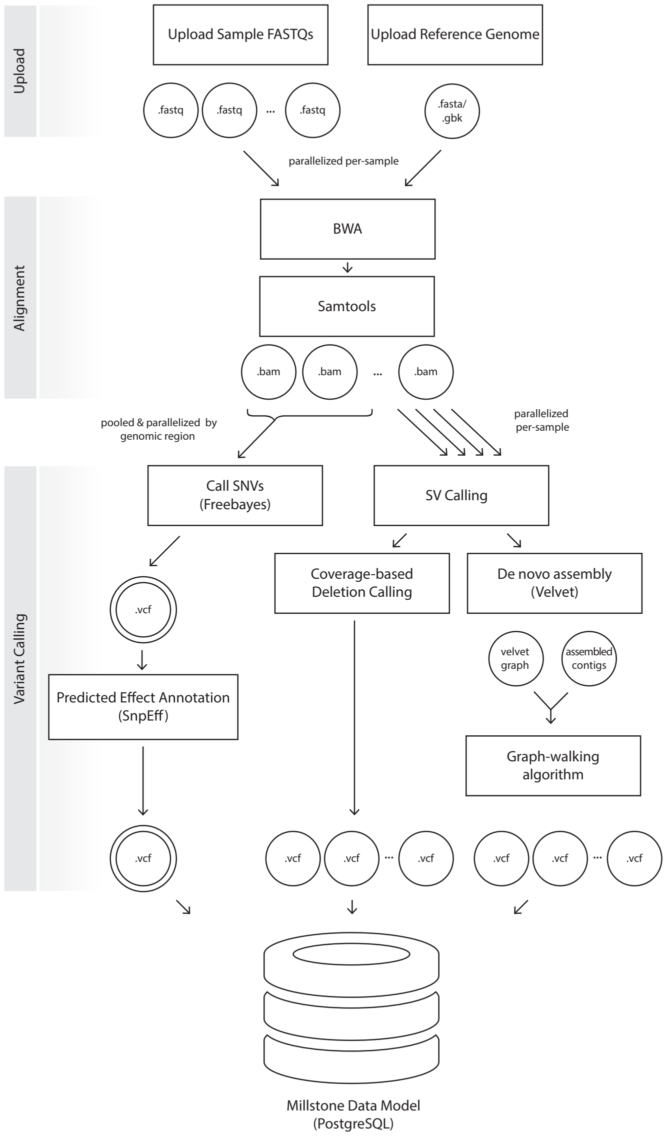

Figure S1: Millstone Analysis Pipeline. The analysis pipeline efficiently automates the process of identifying single nucleotide variants (SNVs) and structural variants (SVs) from user sample data and storing the information in a data model representation that can be explored using Millstone's variant exploration UI. Once sample FASTQs and a reference genome are uploaded, Millstone uses BWA (Li and Durbin 2010) to align samples to the reference, parallelizing alignments across available processor cores. Once all alignments are complete, Freebayes performs SNV-detection on all .bam files simultaneously, parallelizing across regions of the genome. SVs are identified in parallel in individual samples using two methods: 1) Deletions are detected using sequencing read coverage and 2) novel junctions that indicate insertions and rearrangements are identified using *de novo* assembly of unmapped reads using Velvet (Zerbino and Birney 2008) followed by a custom graph-walking algorithm to combine assembled contigs and alignment with BWA to place contigs in the genome. All variant callers report their data in the Variant Call Format (VCF) and Millstone parses the VCFs into a single data model representation. Millstone further uses read coverage to identify regions of the genome with poor mapping quality and automatically adds variants that fall into such regions to appropriate VariantSets.

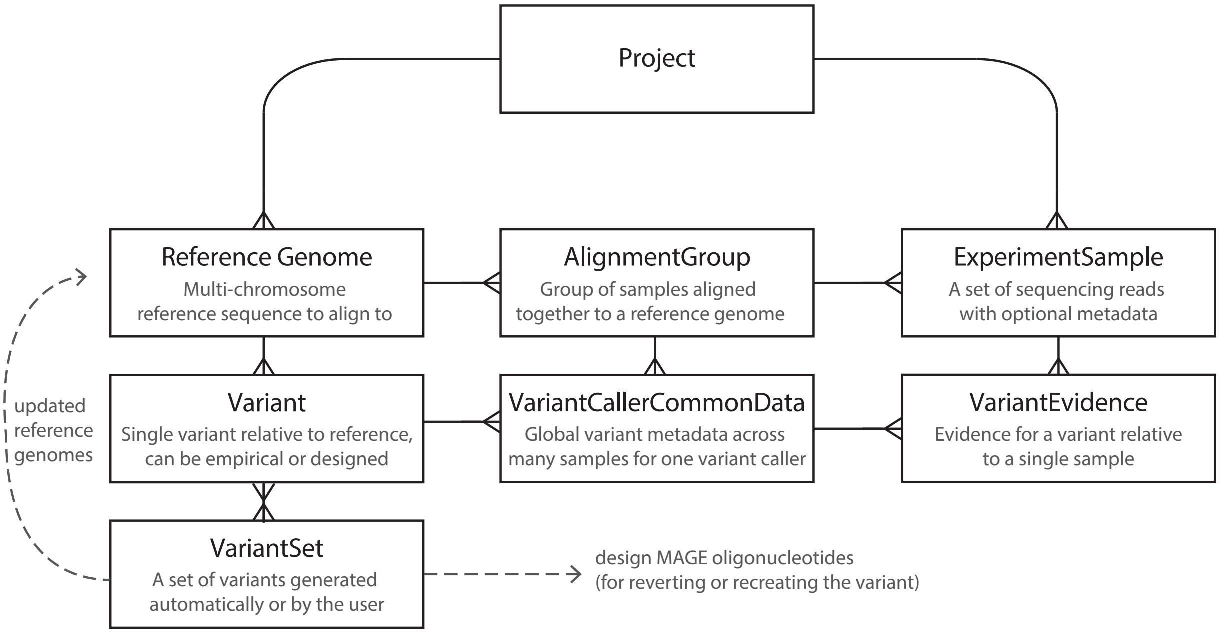

Figure S2: Millstone Data Model. Millstone's data model was designed to enable project organization, data storage, and to support researcher operations including uploading data, running analysis pipelines, exploring the resulting data, and generating actionable outputs. Users upload *ReferenceGenomes* (e.g. Genbank or FASTA genome sequences) and *ExperimentSamples* (e.g. FASTQ) to a Project. The *AlignmentGroup* model stores data from an alignment of multiple *ExperimentSamples* against a specific *ReferenceGenome*. *Variants* represent both user-specified designed mutations and those emprically identified by variant callers. *Variants* are the most important primitive in Millstone, and serve as the unit of operation for analysis and design tasks. *Variants* are defined relative to a specific *ReferenceGenome*. The *VariantCallerCommonData* model relates a given Variant to any *AlignmentGroups* the mutation was called in and stores metadata provided by the variant calling tool (e.g. Freebayes). The *VariantEvidence* model further stores evidence for the occurrence of a specific Variant in a specific ExperimentSample. *VariantSets* allow the user to group Variants and take actions on groups. The VariantSet concept is very similar to tags in other software contexts and a Variant can belong to more than one VariantSet. Operations enabled by VariantSets include filtering in the exploration view, exporting subsets of variants, printing MAGE oligos, and generating new versions of reference genomes. The full data model is declared in the source code: `https://github.com/churchlab/millstone/blob/master/genome_designer/main/models.py`.

a

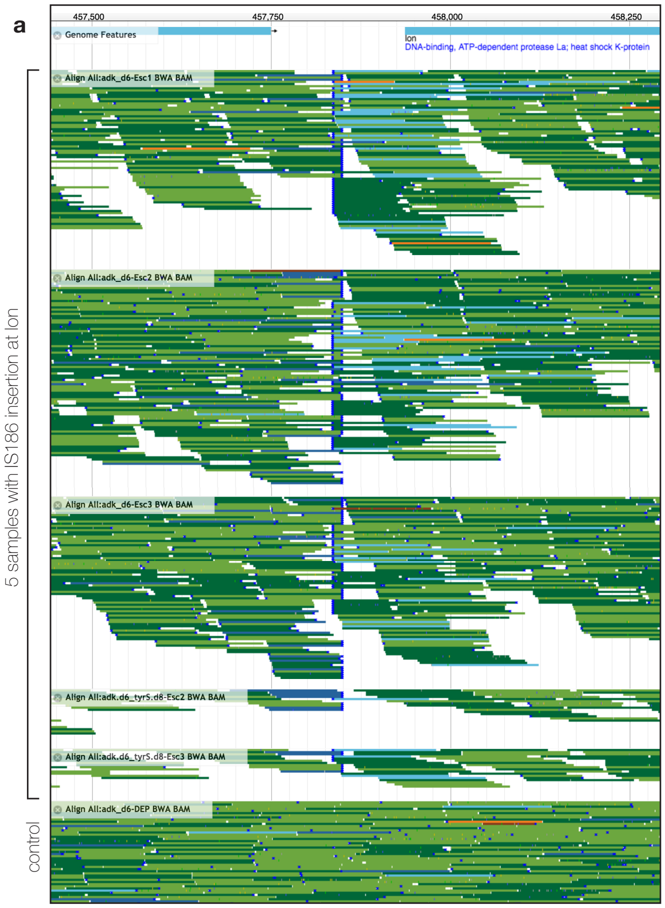

b

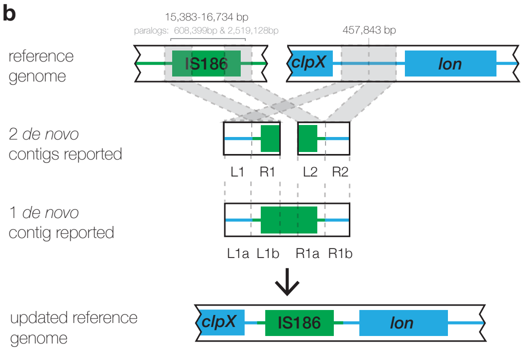

c

Sample	Contig #	Mean Coverage	Contig Size	Left/Right Junction	Genome Position	Gene
adk.d6-Esc3	1569	45.9	360	L1	457848	*lon*
	1569	45.9	360	R1	16733	*IS186*
	673	38.8	532	R2	457835	*lon*
	673	38.8	532	L2	15388	*IS186*
adk.d6-Esc2	571	76.0	2145	L1b	608398	*IS186*
	571	76.0	2145	L1a	457848	*lon*
	571	76.0	2145	R1a	607053	*IS186*
	571	76.0	2145	R1b	457835	*lon*
adk.d6-Esc1	8	38.8	1784	L1b	608399	*IS186*
	8	38.8	1784	L1a	457848	*lon*
	8	38.8	1784	R1a	607053	*IS186*
	8	38.8	1784	R1b	457836	*lon*
adk.d6/tyrS.d8-Esc3	76	9.2	608	L1	457841	*lon*
	76	9.2	608	R1	15385	*IS186*
	2	10.3	481	L2	2519646	*IS186*
	2	10.3	481	R2	457835	*lon*
adk.d6/tyrS.d8-Esc2	4	3.6	236	R1	2519128	*IS186*
	4	3.6	236	L1	457405	*clpX*
	4	3.6	236	L1	456777	*clpX*
	14	6.6	575	L2	16733	*IS186*
	14	6.6	575	R2	457848	*lon*

d

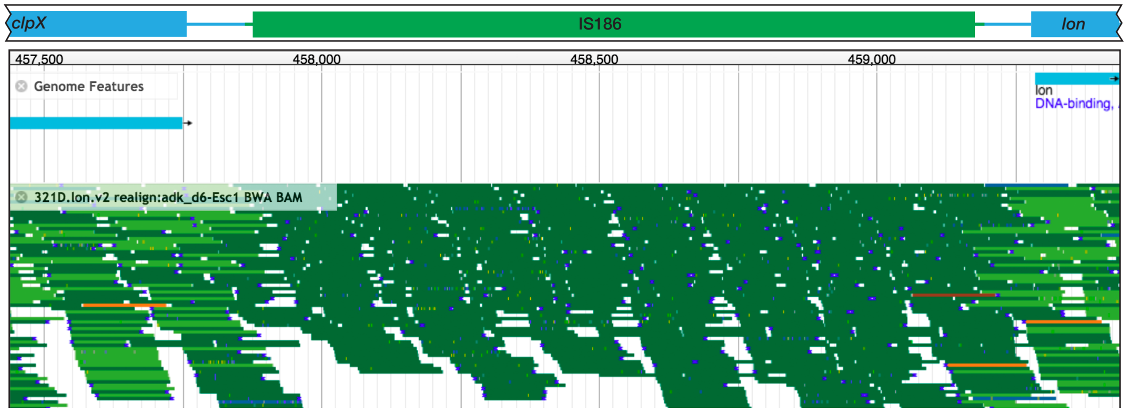

Figure S3: Millstone uses *de novo* assembly to identify a mobile element insertion at the *lon* locus across 5 escapee clones from Mandell *et al.* **(a)** Millstone's integration with JBrowse shows evidence for a disruption upstream of the *lon* gene for 5 escapee strains. Each colored line represents a single read and the read alignments are grouped by sample. A wild-type strain is shown at the bottom for comparison. Darker reads map to the forward strand and lighter reads map to the reverse strand. Properly paired reads are green, reads with an unmapped mate are blue, and reads with improperly paired mates are orange. The dark blue vertical lines denote split reads, indicating a disrupted read alignment. **(b)** Millstone performs *de novo* assembly followed by alignment of assembled contigs back to the reference, and then uses a graph traversal algorithm to identify large insertions. Two separate example cases are shown where either one or two contigs are identified by *de novo* assembly. These contigs are composed of reads that map to the *lon* locus and IS186 mobile element, indicating insertion of IS186 element at the *lon* locus. Finally, Millstone generates an updated reference genome reflecting the insertion. **(c)** A table of contigs, their sizes, and multiple reference alignments for each of the 5 samples with an IS186 insertion. **(d)** A new JBrowse view with the updated reference. The split and mismapping reads are gone, revealing a clean alignment in the region with the inserted IS element. The dark region indicates reads which map to multiple IS186 paralogs across the genome.

Feature	Millstone	BreSeq	SPANDx	Galaxy
Free & Open Source	•	•	•	•
Effect Prediction	•	•	•	•
Variant Visualization	•	•		
Multiple Sample Comparison	•	•	•	
Interactive Querying	•			
Structural Variant Detection	•	•	•	
Genome Versioning	•	•		
Easy Deployment / Install	•	•		•
Genome Editing GUI Workflow	•			
Sharing / Collaboration	•	•		•
Supports Paired-End Data	•		•	•

Table S1: Comparison between Millstone and Other Tools. A tabular comparison of features among different whole-genome sequencing tools, with a focus on those that are meant for use with haploid microbial genomes and are scalable to large datasets. All tools listed here are free and open-source. A more detailed description of the differences between the features and limitations of each is provided in **Supplementary Note 2.** *Effect Prediction:* Prediction of variant effects based on genome annotation. *Variant visualization:* can visualize read alignments for individual variants built into the tool. *Multiple Sample Comparison:* can compare the evidence for and presence/absence of a variant across multiple samples within the tool. *Interactive Querying:* can interactively search and subset variant list based on genomic position, gene, quality, mutation type, etc. within the tool. *Structural Variant Detection:* Supports detection of longer variants not normally detected by SNV callers like Freebayes and GATK Unified Genotyper, such as insertions, deletions, and translocations longer than 50-100 bp in length. *Genome Versioning GUI Workflow:* Capable of generating an updated reference genome based on a subset of variants found in an initial reference genome though an integrated graphical interface (as opposed to command-line tools.) *Easy Deployment / Install:* Can be used without command-line compilation or scripting. *Genome Editing:* Generates designs for iterative editing/reversion of selected variants. In Millstone, a design consists of a set of synthetic or natural variants (i.e. a VariantSet) applied to a reference genome sequence. *Sharing / Collaboration:* Built-in data-sharing via the web among teams of multiple users. *Supports Paired-End Data:* Utilizes paired-end read information to generate alignments and identify structural variants.

	Line	Position	Ref	Alt	Millstone	Tenaillon
**Large Deletions**	LIne142	547700	-	71438 bp	•	
	LIne17	3512040	-	415 bp	•	
	Line106	4499185	-	15462 bp	•	
	Line4	664688	-	1443 bp	•	
**Deletions**	Line74	474295	GATGGTTAATGCC	GC	•	
	Line74	474295	GATGGTTAATGCC	GC	•	
**Insertions**	Line14	2236102	-	9_bp insertion		•
	Line79	474706	-	15_bp insertion		•
	Line106	4090527	-	25_bp insertion		•
	Line18	4089885	TCAGTTTGC	TCAGTTTGCAGTTTGC	•	
	Line58	3571137	TC	TCAGTAC	•	
**Mobile Elements**	Line13	3652063	-	IS150 insertion		•
	Line92	4202813	-	IS186 insertion		•
	Line132	2651161	-	IS150 insertion	•	
	Line112	4376973	-	IS1 insertion	•	
**Point Mutations**	Line7	2031537	G	A	•	
	Line7	2031545	A	T	•	
	Line27	798255	T	C	•	
	Line67	4195430	G	A	•	

Table S2: Variant differences between Millstone and the original analysis performed by Tenaillon et al. We compared variants found by *Tenaillon et al.* to those found automatically Millstone, focusing on Type II errors. Here we split discrepancies into 5 classes, including 3 short nucleotide variant (SNV) classes - short Deletions, Insertions, and Point Mutations, and 2 structural variant classes - Large Deletions and Mobile Element insertions. The final two columns describe 'True Positive' variants which were found by only one of the two pipelines. To identify these, we examined the read evidence using Millstone's JBrowse visualization feature and determined whether the variant was correct as called by either pipeline.

	Biocontainment	GRO	Improving GRO Fitness	Tenaillon
**Number of Genome Samples**	24	68	97	115
**Reference Genome Size**	4.6E6 (+6124 plasmid)	4.6E+06	4.6E+06	4.6E+06
**Mean Aligned Reads per Sample**	2.2E+06	1.2E+07	2.4E+06	5.4E+06
**Alignment + SNV-calling Time**	1hr 12min	5hr 10min	2hr 12min	4hr 35min
**SV calling time**	37min	1hr 30min	50min	30min
**Variants called**	1533	3127	2284	4171
**Average Query Time**	0.5sec	1.5sec	1.4sec	3sec

Table S3: Benchmarking. Millstone's analysis pipeline was executed on datasets of various size from four different projects: Biocontainment (Mandell et al. 2015), GRO (Lajoie et al. 2013), Improving GRO Fitness (*Kuznetsov et al., submitted*), and Tenaillon (Tenaillon et al. 2012). Average query time was calculated using no filter and a simple filter for strong alt calls: `GT_TYPE = 2`. All benchmarking was performed on Amazon Web Services (AWS) Elastic Cloud Compute (EC2) instances r3.8xlarge (32 cores, 244 Gb memory).

.

Supplementary Note 1: Cost of Multiplexed Genome Library Preparation and Sequencing

Sample Preparation. Low-cost high-throughput sample preparation workflows for Illumina sequencing based on transposon insertion and fluorescent dye-based sample quantification can reduce the cost of preparation to below $15 USD per sample and be performed in approximately 5 hours per 96-well plate (Baym et al. 2015).

Multiplexed Illumina Sequencing. For accurate discovery of structural variants, 20-30x coverage is ideal per sample. For the 4.6 megabase *Escherichia coli K12 MG1655* genome at 30x coverage, this is approximately 1 million 150 bp reads. The cost per read for Illumina sequencing can vary widely depending on the platform (NexSeq, MiSeq, HiSeq, etc). As a conservative estimate, a whole HiSeq 2500 lane in Rapid Run mode can produce 250 million paired end reads of 150 base pairs for a cost of approximately $2500 USD, or approximately $10 dollars per bacterial genome. Paired-end 150bp sequencing in Rapid Run mode takes approximately 40 hours. Larger-scale sequencing formats are generally cheaper, but longer to run, and smaller formats, like the MiSeq, are faster, but more expensive per genome.

Supplementary Note 2: Comparison of Millstone to Other Tools

While other packages exist to solve the integration and automation of whole genome resequencing and annotation, most of these tools are built for large diploid genomes, such as *Homo sapiens*. Here we compare features and performance between Millstone and a few other related automated genome re-sequencing tools (see also **Supplementary Table 1**).

Galaxy. Some tools, like Galaxy, allow users to create their own custom pipelines without bash scripting, and do support the creation of pipelines for microbial genomes. However, Galaxy requires that the user to understand and optimize settings for each individual tool. Galaxy also does not allow visualization or interactive querying of the output, and cannot generate new reference genomes or use the output of one round of sequencing to inform the next round. Finally, because of Galaxy's one-size-fits-all nature, optimizing pipeline performance (for example, via inline compression and piping of input and output streams) is challenging.

SPANDx. Another recent tool, SPANDx, can also perform genome resequencing for multiple strains simultaneously, but its widespread use is limited because it can only be run on UNIX computing clusters using the closed-source commercial PBS job scheduling system. SPANDx has no user interface or interactive components, and so users are required to gather the data manually and run the pipeline using a command line interface. Because we could not readily locate a PBS system to test the pipeline on, we were not able to compare the output between SPANDx and Millstone.

breseq. *breseq* is purpose-built to perform haploid genome resequencing, and has become a standard tool for Adaptive Laboratory Evolution experiments (Deatherage and Barrick 2014). *breseq* reports SNV and SV events for single genomes and provides a visualization of raw read evidence for the event. An advantage of Millstone is its ability to perform variant calling on hundreds of genomes in parallel, facilitating analysis of mutation frequency across a population of clones. Millstone's JBrowse integration, data model, and querying features allow researchers to interactively filter, view, and compare genome alignments and variant data (as in **Supplementary Fig. 3**).

Both *breseq* and Millstone use a default variant detection threshold that works well in most cases, and Millstone complements this with an interactive search feature that allows researchers to filter variants after the variant calling pipeline has been run according to characteristics including read depth, number of samples

with the variant, or predicted variant effect.

Millstone further supports paired-end reads, allowing for enhanced detection of structural variation, whereas *breseq* treats paired-end reads as single reads. Millstone can further assemble and place *de novo* contigs onto existing reference genomes. Millstone can be used pre-configured on Amazon Web Services (AWS) and so does not require proficiency with UNIX nor the manual installation of dependencies. Millstone's user interface also automates the process of copying potentially large amounts of whole-genome data to the remote server.

References

Baym, Michael et al. (2015). “Inexpensive Multiplexed Library Preparation for Megabase-Sized Genomes”. In: *bioRxiv*, p. 013771.

Deatherage, Daniel E and Jeffrey E Barrick (2014). “Identification of mutations in laboratory-evolved microbes from next-generation sequencing data using breseq”. In: *Engineering and Analyzing Multicellular Systems: Methods and Protocols*, pp. 165–188.

Lajoie, Marc J et al. (2013). “Genomically recoded organisms expand biological functions”. In: *Science (New York, NY)* 342.6156, pp. 357–360.

Li, Heng and Richard Durbin (2010). “Fast and accurate long-read alignment with Burrows–Wheeler transform”. In: *Bioinformatics* 26.5, pp. 589–595.

Mandell, Daniel J et al. (2015). “Biocontainment of genetically modified organisms by synthetic protein design”. In: *Nature* 518.7537, pp. 55–60.

Tenaillon, O et al. (2012). “The Molecular Diversity of Adaptive Convergence”. In: *Science (New York, NY)* 335.6067, pp. 457–461.

Zerbino, Daniel R and Ewan Birney (2008). “Velvet: algorithms for de novo short read assembly using de Bruijn graphs”. In: *Genome research* 18.5, pp. 821–829.
